# Establishment and validation of novel MRI radiomic feature-based prognostic models to predict progression-free survival in locally advanced rectal cancer

**DOI:** 10.3389/fonc.2022.901287

**Published:** 2022-11-03

**Authors:** Fei Xie, Qin Zhao, Shuqi Li, Shuangshuang Wu, Jinli Li, Haojiang Li, Shenghuan Chen, Wu Jiang, Annan Dong, Liqing Wu, Long Liu, Huabin Huang, Shuoyu Xu, Yuanzhi Shao, Lizhi Liu, Li Li, Peiqiang Cai

**Affiliations:** ^1^ Sun Yat-sen University Cancer Center, State Key Laboratory of Oncology in South China, Collaborative Innovation Center for Cancer Medicine, Guangdong Key Laboratory of Nasopharyngeal Carcinoma Diagnosis and Therapy, Guangzhou, Guangdong, China; ^2^ School of Physics, State Key Laboratory of Optoelectronic Materials and Technologies, Sun Yat-sen University, Guangzhou, China; ^3^ The Second Affiliated Hospital of Guangzhou University of Chinese Medicine, Guangzhou, China; ^4^ Department of Radiology, The Sixth Affiliated Hospital of Guangzhou Medical University, Qingyuan People’s Hospital, Qingyuan, China; ^5^ Department of Radiology, Guangzhou Concord Cancer Center, Guangzhou, China; ^6^ Department of Radiology, The Seventh Affiliated Hospital of Sun Yat-sen University, Shenzhen, China; ^7^ Department of Radiology, The Third People’s Hospital of Shenzhen, Shenzhen, China

**Keywords:** magnetic resonance imaging, radiomics, prognostic models, locally advanced rectal cancer, clinical predictors

## Abstract

In locally advanced rectal cancer (LARC), an improved ability to predict prognosis before and after treatment is needed for individualized treatment. We aimed to utilize pre- and post-treatment clinical predictors and baseline magnetic resonance imaging (MRI) radiomic features for establishing prognostic models to predict progression-free survival (PFS) in patients with LARC. Patients with LARC diagnosed between March 2014 and May 2016 were included in this retrospective study. A radiomic signature based on extracted MRI features and clinical prognostic models based on clinical features were constructed in the training cohort to predict 3-year PFS. C-indices were used to evaluate the predictive accuracies of the radiomic signature, clinical prognostic models, and integrated prognostic model (iPostM). In total, 166 consecutive patients were included (110 vs. 56 for training vs. validation). Eleven radiomic features were filtered out to construct the radiomic signature, which was significantly related to PFS. The MRI feature-derived radiomic signature exhibited better prognostic performance than the clinical prognostic models (*P* = 0.007 vs. 0.077). Then, we proposed an iPostM that combined the radiomic signature with tumor regression grade. The iPostM achieved the highest C-indices in the training and validation cohorts (0.942 and 0.752, respectively), outperforming other models in predicting PFS (all *P* < 0.05). Decision curve analysis and survival curves of the validation cohort verified that iPostM demonstrated the best performance and facilitated risk stratification. Therefore, iPostM provided the most reliable prognostic prediction for PFS in patients with LARC.

## Introduction

More than 100,000 cases of rectal cancer are diagnosed worldwide annually, and approximately 70% are locally advanced rectal cancer (LARC) (1). The standard treatment for LARC is neoadjuvant chemoradiotherapy (nCRT) followed by surgery (2, 3). Adjuvant chemotherapy is recommended for all patients with stage II/III rectal cancer after neoadjuvant radiochemotherapy and surgery (4). However, the effect of postoperative chemotherapy on survival remains controversial (5, 6). Therefore, pre- and post-treatment prediction models are needed to help determine treatment strategies and identify patients who may benefit from postoperative adjuvant chemotherapy.

LARC prognosis is based on the tumor–node–metastasis (TNM) staging system. Recently, nomograms were proposed to improve LARC prognosis prediction (7–10), using indexes such as age, carcinoembryonic antigen (CEA), carbohydrate antigen 19–9 (CA19-9), pathological tumor stage (ypT), pathological nodal stage (ypN), and tumor regression grade (TRG). However, contradictory results have been obtained with these models. For example, in one study, TRG was more efficient than ypTN stage in predicting the outcome (11), whereas in another study, ypTN stage contributed more (10), indicating that these nomograms are not robust enough for clinical application.

Radiomics, a tool that reveals underlying tumor heterogeneity using medical images (12, 13), serves as a strong prognostic predictor for malignancies (14). In rectal cancer, radiomics based on magnetic resonance imaging (MRI) is highly efficient in evaluating the tumor response to nCRT (14, 15) and can help identify non-responders (16) and pathologic complete responders (17–20). Prediction models based on radiomic features have added predictive ability in combined models, enhancing the accuracy by up to 74% (sensitivity 58%, specificity 77%) (18). Combined models based on radiomics and clinical data can independently predict overall survival (21), disease-free survival (22–24), and progression-free survival (PFS) (15) in LARC. However, pretreatment radiomics only reflects the cancer characteristics before surgery. Considering effective treatments may revise radiomics features, a study innovatively used delta radiomics based on 4 features to predict distant metastasis (DM) in LARC, obtaining a test set balanced accuracy, sensitivity and specificity of 78.5%, 71.4% and 85.7%, respectively (25). However, other prognostic clinical and histological indicators, including TRG, associated with the efficacy of nCRT on long term survival, which is important for determining the follow-up treatment after surgery, have not been considered in the radiomic prediction model. Thus, whether the combined model with radiomics and postoperative TRG data has an improved predictive ability for risk classification remains to be determined.

Here, we investigated the abilities of prognostic models based on radiomics, pre- and post-treatment clinical factors, and combination of radiomics and pre- and post-treatment clinical factors for predicting 3-year early PFS in LARC. Furthermore, we explored the internal correlations and differences among the models to determine the effect of combining different types of markers.

## Methods

### Patient selection

A total of 166 patients with LARC, based on pathological examination between March 2014 to August 2016, were enrolled (**Supplementary Figure S1**). The inclusion criteria were: (1) age ≥ 18 years; (2) newly diagnosed with LARC (staged on MRI as cT2–4 and/or N+) without distant metastasis and other malignancies; (3) treatment with preoperative nCRT; (4) high resolution pelvic MRI examination before nCRT; and (5) availability of complete electronic medical records and imaging data. Patients who did not complete nCRT were excluded. Considering only using radiomics feautures from pretreatment, pelvic MRI after treatment was not mandatory. Basic clinical information (sex, age, weight, height, BMI, clinical T stage, and clinical N stage) and laboratory indicators (routine blood tests, liver and renal function tests, blood glucose monitoring, C-reactive protein, serum lipid level, CEA, and CA19-9 levels) were collected before nCRT. This study was approved by the institutional ethics committee of our hospital. As this study was retrospective, the requirement for informed consent was exempted.

### Treatment and follow-up

The preoperative treatment regimen included intensity-modulated radiation therapy (IMRT) and concurrent chemotherapy. IMRT doses of 50 Gy for gross tumor volume (primary tumors and enlarged LNs) and 45 Gy for clinical target volume were divided into 25 fractions. Two different chemotherapy regimens were used. Of the 166 patients, 91 received oxaliplatin (OXA) and capecitabine (CAPOX) for 3 weeks; 130 mg/m² OXA was intravenously administered on the first day, and 1,000 mg/m² capecitabine (CAP) was orally administered twice a day for the first two weeks. The remaining 75 patients received oral administration of 1,000 mg/m² CAP twice a day for the first 14 days. Radical rectal resection was performed 6–8 weeks after the completion of nCRT. After surgery, ypT stage, ypN stage, and TRG were evaluated.

The PFS, calculated at the endpoint, was defined as the interval from surgery to tumor progression, including local recurrence and/or metastasis or death. Follow-up visits were performed every 3–6 months in the first 2 years, then every 6 months in the following 3 years, and once a year thereafter.

### MRI scanning and segmentation

Pretreatment pelvic MRI was performed using Trio Tim 3.0T (n = 74; Siemens Healthcare GmbH Henkestr) with two body Matrix coils and two spine Matrix coils or Discovery750 3.0T (n = 92; GE Healthcare) using an 8-channel phased array body coil in the supine position. Gadolinium-diethylenetriamine pentaacetic acid was injected as the contrast agent at a dosage of 0.1 mL/kg with a flow velocity of 3.0 mL/s. The scanning protocol included the axial, coronal, and sagittal T1-weighted (T1-w) images; T2-weighted (T2-w) images, axial short-axis T2-weighted images (short-axis T2-w), and contrast-enhanced T1-weighted (T1C-w) sequences. Short-axis T2–w, a thin section (3 mm) T2-weighted fast spin echo sequence acquired in a plane perpendicular to the long axis of the tumor (26), was helpful to precisely examine the tumor and its relationship with the intestinal wall, mesorectal fascia, vessels, and adjacent organs.

MR images were retrieved from the picture archiving and communication system and loaded onto AnalyzePro^1^ for manual segmentation. Two radiation oncologists with more than 10 years of experience outlined the whole-tumor volumes of interest, representing the contour of the tumor on each slice of all sequences separately.

### Extraction of radiomic features and radiomic signature construction

Feature extraction in this study was performed using PyRadiomics^2^ (27). In total, 14,089 radiomic features were extracted from the axial T1C-w, T1-w, T2-w, and short-axis T2-w scans. The parameter settings for image preprocessing and radiomic feature extraction are presented in the **Supplementary Information**. To evaluate the effect of semi-automatic segmentations on the values of radiomic features, the inter-class correlation coefficient (ICC) was utilized. For this, 30 patients in the training cohort were randomly selected and segmented by two other radiation oncologists with more than 10 years of experience (**Supplementary Figure S2A**). The stability of each extracted feature was assessed by different expert radiologists. Stable radiomic features were defined as ICCs > 0.8 (**Supplementary Figure S2B**).

The RAD score was computed for each patient using a linear combination of selected features, weighted by their respective coefficients, and used to construct a radiomic signature. The potential association of the radiomic signature with 3-year early PFS was evaluated in the training cohort and then validated in the validation cohort.

### Statistical analysis

The LASSO Cox regression method (**Supplementary Information**) was applied to select the most effective combination of prognostic features. The models were determined using the backward stepwise Akaike information criterion method, in which the least significant variables were removed one by one after fitting a full model with the candidate variables. The Mann-Whitney U test for continuous variables and the chi-square test for categorical variables were used to compare clinical characteristics between the training and validation cohorts. Considering multiple factors (including clinical T stage, clinical N stage, TRG, CEA, CA19-9, GLO, ypT stage, ypN stage), univariate and multivariate analyses were performed with the Cox proportional hazards model, and the hazard ratios (HRs) and 95% confidence intervals (CIs) were calculated. Harrell’s concordance indices were used to assess the predictive power of each model. Statistical analyses were performed using R version 4.0.2^3^. R packages, including glmnet, caret, survival, rms, Hmisc, corrplot, pheatmap, and rmda, were used. All statistical tests were two-sided, and *P* < 0.05 was considered statistically significant.

## Results

A total of 166 consecutive patients (median age, 58 [interquartile range, 49‒64] years; 117 [70.48%] men) were included in the analysis. The patients were randomly divided in a 2:1 ratio, with 110 in the training cohort and 56 in the validation cohort. **Table 1** summarizes the clinical characteristics of the patients with LRAC in the training and validation cohorts. There were no differences in those characteristics between the training and validation cohorts (*P* = 0.202–0.930).

**Table 1:** Clinical and pathological characteristics of patients in the training and validation cohorts.

	Total	Training cohort	Validation cohort	*P*
Characteristics	(N = 166)	(N = 110)	(N = 56)	values
	No. (%)	No. (%)	No. (%)	
Gender				0.582
Female	49 (29.52)	34 (30.91)	15 (26.79)	
Male	117 (70.48)	76 (69.09)	41 (73.21)	
Age (years)				
Median (IQR)	58 (49-64)	58 (49-64)	57 (49-64)	0.792
TRG				0.514
1	41 (24.70)	30 (27.27)	11 (19.64)	
2	48 (28.92)	28 (25.45)	20 (35.71)	
3	53 (31.93)	36 (32.73)	17 (30.36)	
4	24 (14.46)	16 (14.55)	8 (14.29)	
Clinical T stage				0.202
T2	9 (5.42)	7 (6.36)	2 (3.57)	
T3	109 (65.66)	76 (69.09)	33 (58.93)	
T4	48 (28.92)	27 (24.55)	21 (37.50)	
Clinical N stage				0.511
N0	41 (24.7)	28 (25.45)	13 (23.21)	
N1	56 (33.73)	40 (36.36)	16 (28.57)	
N2	69 (41.57)	42 (38.18)	27 (48.21)	
ypT stage				0.311
T0	33 (19.88)	26 (23.64)	7 (12.50)	
T1	19 (11.45)	12 (10.91)	7 (12.50)	
T2	55 (33.13)	38 (34.55)	17 (30.36)	
T3	51 (30.72)	29 (26.36)	22 (39.29)	
T4	8 (4.82)	5 (4.55)	3 (5.36)	
ypN stage				0.930
N0	135 (81.33)	90 (81.82)	45 (80.36)	
N1	27 (16.27)	17 (15.45)	10 (17.86)	
N2	4 (2.41)	3 (2.73)	1 (1.79)	
CEA				0.505
≤ 5ng/ml	89 (53.61)	61 (55.45)	28 (50.00)	
> 5ng/ml	77 (46.39)	49 (44.55)	28 (50.00)	
CA19-9				0.496
≤ 35U/ml	138 (83.13)	93 (84.55)	45 (80.36)	
> 35U/ml	28 (16.87)	17 (15.45)	11 (19.64)	
Treatment				0.921
CAP	75 (45.18)	50 (45.45)	25 (44.64)	
CAPOX	91 (54.82)	60 (54.55)	31 (55.36)	
Follow-up time (month)				
Median (IQR)	36.3 (28.1-43.6)	36.1 (29.0-43.8)	37.0 (25.9-43.6)	0.870

Data are n (%) unless otherwise indicated. P values were calculated by the Mann-Whitney U test for continuous variables and Chi-square test for categorical variables. No significant differences were found between the training cohort and the validation cohort (P = 0.202–0.930).

IQR, inter-quartile range; TRG, tumor regression grade; ypT/N stage, the pathologic classification after nCRT; CEA, carcinoembryonic antigen; CA19-9, carbohydrate antigen 19-9; CAPOX, oxaliplatin and capecitabine chemotherapy; CAP, capecitabine chemotherapy.

The median follow-up duration was 32.3 months (range, 2.6–58.8 months). During the last follow-up, disease progression was confirmed in 24 patients (21.8%) in the training cohort and 14 (25.0%) in the validation cohort (*P* = 0.708).

After univariate analysis in the training cohort, candidate variables with a *P* value < 0.1 were included in the multivariate model analysis. Pretreatment variables, including clinical T stage, CEA, CA 19-9, and globulin, and post-treatment predictors, including pathologic T stage and TRG, were selected as summarized in **Supplementary Table S1**. The pretreatment clinical prognostic model (PreM) for 3-year PFS prediction was established based on the four pretreatment variables. Multivariate analysis identified CEA and globulin as independent predictors (**Supplementary Table S2**). The clinical stage prognostic model was built based on the clinical T and N stages.

The C-indices of PreM and the clinical stage prognostic model were 0.627 (95% confidence interval [CI]: 0.572–0.682) and 0.578 (95% CI: 0.522–0.634) in the training cohort, and 0.552 (95% CI: 0.482–0.622) and 0.611 (95% CI: 0.531–0.691) in the validation cohort, respectively (**Table 2**). The nomograms and corresponding calibration curves for the probability of 3-year PFS generated using the pretreatment clinical prognostic models are shown in **Supplementary Figure S3**.

**Table 2:** C-index for each prognostic model for survival prediction.

Models for survival prediction		Predictors in each model	Training cohort		Validation cohort					
			C-index	95% CI	C-index	95% CI	*P* values			
Pretreatment clinical prognostic models	cTN	cT + cN	0.578	(0.522-0.634)	0.611	(0.531-0.691)	.429	.503	.039*	<.001*
	PreM	CEA+GLO	0.627	(0.572-0.682)	0.552	(0.482-0.622)	.307	.356	.007*	<.001*
Posttreatment clinical prognostic models	ypTN	ypT+ypN	0.675	(0.615-0.735)	0.532	(0.441-0.623)	.156	.636	.033*	<.001*
	PostM1	CEA+GLO+ypT	0.737	(0.679-0.795)	0.603	(0.534-0.672)	*ref*	.647	.027*	<.001*
	PostM2	TRG+CEA+GLO	0.664	(0.603-0.725)	0.609	(0.542-0.676)	.647	*ref*	.077	.009*
Radiomics signature	Radscore	Radscore	0.937	(0.917-0.957)	0.730	(0.651-0.809)	.010*	.077	*ref*	.014*
Integrated prognostic model	iPostM	Radscore+TRG	0.942	(0.922-0.962)	0.752	(0.684-0.820)	<.001*	.009*	.014*	*ref*

P values were calculated by comparing with the corresponding reference prognostic model in each column in the validation cohort (ref represents the reference model). A P value < 0.05 indicates a significant difference.

*Represent P < 0.05. In the validation cohort, five clinical prognostic models showed similar PFS predictive power. Notably, compared with PreM without TRG, the constructed PostM2 achieved better predictive performance (P = 0.356). The developed radiomics signature appeared to be more accurate than clinical prognostic models (P = 0.007 to 0.077). The integrated model (iPostM) combining radiomics signature and TRG gained the highest C-index in the validation cohort (0.752), outperforming the radiomics signature and all other clinical prognostic models in term of evaluating 3-year PFS (all P < 0.05).

cTN, the clinical stage prognostic model; PreM, the pre-treatment clinical prognostic model; ypTN, the pathologic stage prognostic model; PostM1, the post-treatment clinical prognostic model; PostM2, the post-treatment clinical prognostic model without pathologic stage; iPostM, the integrated prognostic model combining TRG and radiomics signature; CI, confidence interval; GLO, globulin; TRG, tumor regression grade; cT/N: clinical T/N stage; ypT/N, the pathologic classification after nCRT; CEA, carcinoembryonic antigen; CA19-9, carbohydrate antigen 19-9.

**Figure 1:**
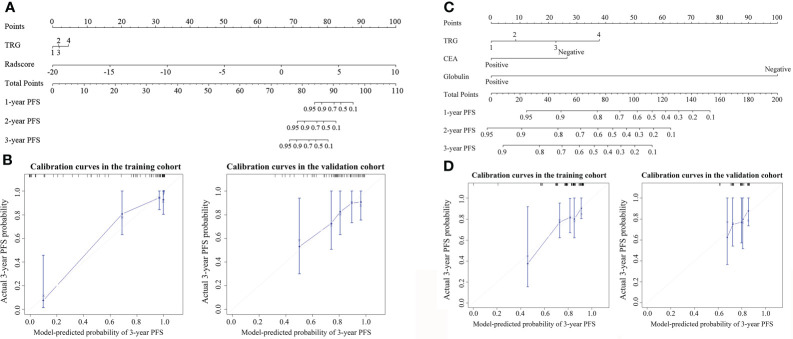
Nomogram for 3-year progression-free survival (PFS) in **(A)** iPostM and **(C)** PostM2. The nomogram allows the user to determine the probability of 3-year PFS corresponding to a patient’s combination of covariates. Calibration curves for predicting 3-year PFS in **(B)** iPostM and **(D)** PostM2 in the training and validation cohorts. The y-axis shows observed survival estimated using the Kaplan-Meier method, and the x-axis shows predicted survival calculated using the prognostic model. The closer fit to the diagonal dotted line indicates a better assessment. iPostM, integrated prognostic model combining tumor regression grade (TRG) and radiomic signature; PostM2, post-treatment clinical prognostic model without pathologic stage.

Additionally, by integrating the clinical variables for pre- and post-treatment, we established post-treatment prognostic models. The pathologic stage prognostic model was built based on the pathologic T and N stages. Stepwise multivariable analyses identified pathologic T stage, CEA, and globulin as independent predictors for PFS in the first post-treatment prognostic model (PostM1). In the validation cohort, PostM1 showed a higher predictive power for PFS than PreM (*P* = 0.307) and the pathologic stage prognostic model (*P* = 0.156), but it was slightly lower than that of the clinical stage prognostic model (*P* = 0.429). When the influences of clinical T stage and pathologic T stage were excluded, TRG, CEA, and globulin remained significant for PFS after performing multivariate Cox regression. Consequently, PostM2 was built with these three factors. The C-indices of the three post-treatment prognostic models are summarized in **Table 2**. The nomograms and corresponding calibration curves for the 3-year PFS probability generated using the post-treatment clinical prognostic models are shown in **Figure 1** and **Supplementary Figure S4**. The multivariable Cox regression results of the pre- and post-treatment clinical prognostic models are summarized in **Supplementary Table S2**. Notably, compared with PreM without TRG, PostM2 achieved a better predictive performance (*P* = 0.356), which indicates that TRG is an important prognostic factor for predicting PFS.

In the training cohort, we selected 11 radiomic features based on MRI that were significantly associated with PFS (**Supplementary Table S3**). The detailed selection process and LASSO results are shown in **Supplementary Figures S5 and S6**, respectively. The formula for the Rad score calculation of the radiomic prognostic model is shown in the **Supplementary Information**. In the training cohort, the radiomic signature yielded a C-index of 0.937 (95% CI: 0.917–0.957). The good prognostic performance of this radiomic signature was validated with a corresponding C-index of 0.730 (95% CI: 0.651‒0.809) in the validation cohort. The radiomic nomogram showed significant improvement compared to the clinical prognostic models (*P* = 0.007‒0.039), except for PostM2 (*P* = 0.077) (**Table 2**). Thus, the developed radiomic signature was more accurate than the clinical prognostic models for evaluating 3-year PFS.

Next, we built two integrated prognostic models that combined the radiomic signature based on MRI features with important pre- and post-treatment clinical factors. Using the multivariate Cox proportional hazard model based on pretreatment clinical factors and radiomic signature, we found that only the radiomic signature remained significant for PFS after adjusting for various cofactors. The integrated PostM (iPostM) was constructed using the radiomic signature and TRG (**Table 3**). The iPostM showed significant improvement compared to the radiomic signature in terms of evaluating 3-year PFS (C-index: 0.752; 95% CI: 0.684‒0.820), with a *P* value < 0.05 (**Table 2**).

**Table 3:** Multivariate Cox regression analysis of the final integrated model.

Variables	Coefficient	HR (95% CI)	*P* values
Radiomics signature			
(per 1 increase)	1.029	2.797 (1.934-4.046)	4.74E-08
TRG			
1			
2	0.543	1.721 (0.555-5.339)	0.347
3	0.551	1.735 (0.445-6.758)	0.427
4	1.417	4.125 (1.043-16.303)	0.043

Hazard ratios estimated by Cox proportional hazards regression. All statistical tests were two-sided. The results of the multivariate Cox analysis correspond to the nomogram in **Figure 1A**.

HR, hazard ratio; CI, confidence interval; TRG, tumor regression grade.

The result of iPostM is visually represented by a nomogram, as shown in **Figure 1A**. The calibration curve for the 3-year PFS probability showed good agreement between the evaluation based on nomogram and actual survival (**Figure 1B**).

**Figure 2:**
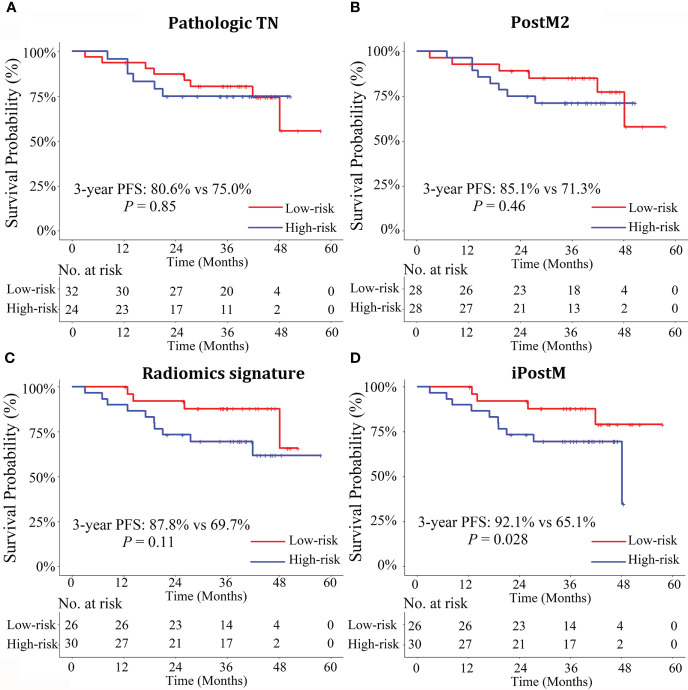
Stratified Kaplan-Meier analyses of the prognostic models to estimate 3-year progression-free survival (PFS) in various risk stratification subgroups in the validation cohort. Patients with high and low risks of PFS were stratified by the prognostic models **(A)** Pathologic TN, **(B)** PostM2, **(C)** Radiomics signature, **(D)** iPostM models. Only the iPostM could stratify patients into high- and low-risk groups based on significantly different 3-year PFS rates (P < 0.05). The log-rank test was used to calculate P values. Pathologic TN, pathologic stage prognostic model; PostM2, post-treatment clinical prognostic model without pathologic stage; iPostM, integrated prognostic model combining tumor regression grade (TRG) and radiomic signature.

Then, we calculated the risk scores of all the prognostic models for each patient in both the training and validation cohorts and then classified the patients into categories: low-risk (patients with a score < 0) and high-risk (patients with a score ≥ 0), with zero as the risk score cutoff. The survival curves between patients in the low- and high-risk categories, generated using clinical and radiomic prognostic models in the training and validation cohorts, are shown in **Supplementary Figures S7** and **S8**, and **Figure 2**. The patients with disease progression after treatment were concentrated in the high score area, and the survival curve showed good prognostic stratification of patients in the low-and high-risk groups in the validation cohort of iPostM. However, such trends were not observed in the validation cohort of other clinical prognostic models and radiomic signature.

**Figure 3:**
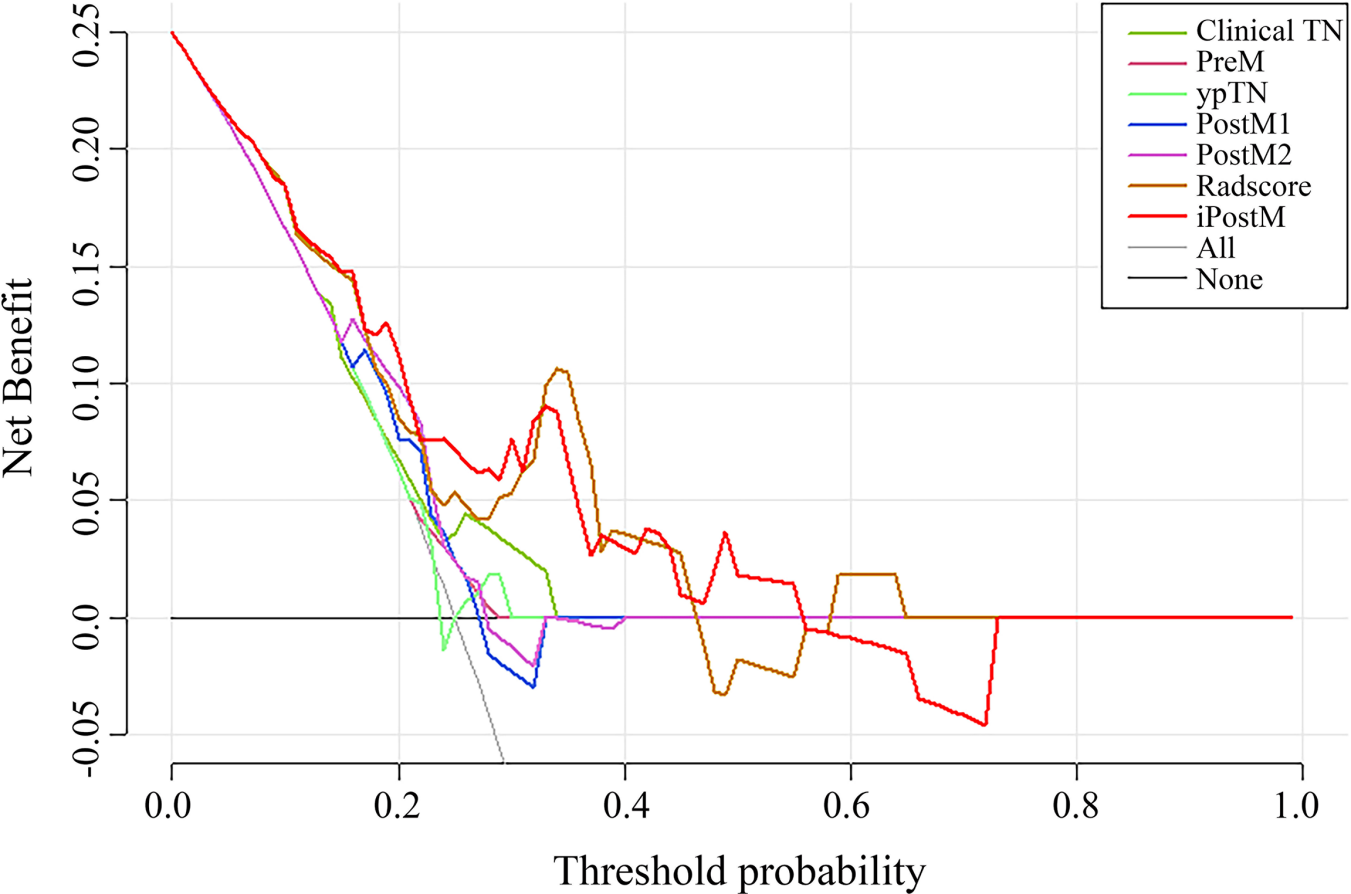
The DCA curves of the nomograms compared for 3-year progression-free survival (PFS) in the validation cohort. The x-axis represents the threshold probabilities, and the y-axis indicates the net benefit. The net benefit is calculated by adding the benefits (true-positive results) and subtracting the risks (false-positive results), with the latter weighted by a factor related to the harm of an undetected cancer relative to the harm of unnecessary treatment. Clinical TN, clinical stage prognostic model; PreM, pretreatment clinical prognostic model; ypTN, pathologic stage prognostic model; PostM1, post-treatment clinical prognostic model; PostM2, post-treatment clinical prognostic model without pathologic stage; iPostM, integrated prognostic model combining tumor regression grade (TRG) and radiomic signature.

Decision curve analysis of the validation cohort in all the prognostic models showed that iPostM was the most efficient (**Figure 3**). The iPostM exhibited the highest efficacy especially in high risk areas. Using a heatmap to determine the association between the radiomic signature and clinical data, we found no significant correlation between the radiomic signature and clinical factors (**Supplementary Figure 9**). In contrast, TRG and pathologic T stage showed a strong correlation (r = 0.69).

## Discussion

In the present study, we established effective prognostic models for predicting 3-year PFS in patients with LARC. Further, we proposed an integrated prognostic model that combined the radiomic signature and TRG and yielded the highest C-index with a value of 0.752 in the validation cohort, outperforming the radiomic signature and all other clinical prognostic models. There was a strong correlation between TRG and pathologic T stage (r = 0.69). Notably, only the integrated post-treatment prognostic model could stratify patients into high- and low-risk groups based on significantly different 3-year PFS rates.

The prognostic models, based on only clinical and pathological factors, showed relatively weak predictive performance, indicating the need to find more useful markers. Additionally, the C-indices of PostMs were higher than those of PreM, possibly because pathological information can better reflect the preoperative state of the tumor. The models with TRG, used as a qualitative evaluation of tumor cells replaced by fibrosis that can reflect the sensitivity of tumors to nCRT, showed higher C-indexes, confirming the findings of previous studies (11, 28–30). TRG was significantly correlated with ypT, which may lead to the absence of ypT in multi-parameter models. Different from some previous studies (10, 24, 31), the discriminatory power of ypN was weakened and not included in the final model, which may attributed to the heterogenous distribution of ypN stage, the adding of TRG and radiomics in our model. For example, Cui et al. (24) included ypN in the prediction model instead of TRG. In their study, the percentages of ypN 0 in the training and validation groups were 60.3% and 50.9%, respectively, which were lower than those in our study (81.33% and 80.36%) and previous findings (96.9% and 94.6%) (9). Thus, to make radiomics-based model more robust, larger sample size and external validation are needed to confirm the results.

Our study indicates that radiomics is an independent prognostic factor for predicting 3-year early PFS in LARC, which is in accordance with a previous study (23). MRI is the standard imaging method for post-nCRT evaluation in LARC, and MRI-based radiomics, can provide minable information from conventional medical images and dig out quantitative features which can reflect tumor heterogeneity, other intrinsic characteristics and microenvironment related with individualized biological behavior of tumor (12–14, 25, 32). It is reported that higher levels of radiomics heterogeneity (ie, higher entropy) was associated with worse response to treatment and/or survival (33, 34), which may caused by the occurrence of constant complex mutations within a tumor to remain resistant to treatment (34). The correlation coefficient between the RAD score and clinical data was low, indicating that radiomic features include details derived from images rather than the clinical TNM stage derived from the macro level (35). Further, compared clinical and pathological models with underfitting, the iPostM obtained enough fitting in training cohort and achieved the highest C-index in the validation cohort, in which the RAD score contributed significantly to the nomogram. The reason may be that the RAD score comprises multiple underlying tumor characteristics associated with disease risks (32, 36, 37), whereas TRG is merely a pathological signature. Additionally, radiomics may decrease the discriminatory power of previously proven independent prognostic indicators (8, 38, 39) as shown in **Supplementary Table S4**. Thus, radiomics was the most influential indicator in the nomogram, followed by TRG and others. Based on the relatively comprehensive information, iPostM may identify patients in high-risk groups and suggest the administration of adjuvant chemotherapy after TME (4) to reduce the risk of occurrence of distant metastasis and recurrence.

Based on the strengths of similar prognostic prediction models for LARC (23, 24), we included multiparametric MR images for extracting radiomic features, with the addition of T1-w, T2-w, and short-axis T2-w, while other studies included relatively fewer sequences. Multiple MRI sequences can detect anatomical details and provide more specific histological information, such as necrosis, cystic degeneration, hemorrhage, and tumor angiogenesis (40). Notably, previous studies have proven that the predictive performance of a radiomic model derived from multi-modal MRI is superior to that based on mono-modal MRI (8, 38). Hence, our model could improve the prognosis ability for patients with LARC.

Nevertheless, there are some limitations to our study. First, it was a relatively small retrospective study performed and validated at a single center with short follow-up duration; however, we enrolled the patients consecutively to reduce underlying selective bias. Second, we only extracted signatures from preoperative primary tumors, lacking lymph nodes, functional MRI images, such as diffusion weighted imaging and apparent diffusion coefficient, which would provide more signatures and inner information. In addition, the value of post-treatment radiomics features need to be further confirmed. Third, we focused on random combinations of imaging features with clinical data rather than genetic heterogeneity. Furthermore, TRG, which is included in our best performing model, was not available to predict the prognosis for the “wait and see” patients. Future studies need to be carried out to validate the prognostic value of our iPostM model in multiple centers with longer follow-up duration.

In summary, we developed pre- and post-treatment prediction models based on clinical and radiomic features. Post-treatment prognostic models with postoperative pathological factors showed better predictive performance than pretreatment prognostic models, and TRG was important for predicting the 3-year PFS of LARC. The multi-modal MRI radiomic model with improved predictive ability could act as a pretreatment-independent prognostic factor for LARC and assist clinicians in determining appropriate neoadjuvant chemoradiotherapy regimens. In addition, the integrated post-treatment prognostic model has potential for recognizing high-risk patients who may benefit from postoperative adjuvant therapy. In future studies, we will validate the performance of our model and explore its clinical applications.

## Data availability statement

The raw data supporting the conclusions of this article will be made available by the authors, without undue reservation.

## Ethics statement

The studies involving human participants were reviewed and approved by the institutional ethics committee of Sun Yat-sen University Cancer Center. Written informed consent for participation was not required for this study in accordance with the national legislation and the institutional requirements.

## Author contributions

Conception and design of this study: FX, QZ and SL. Patients providing: FX, QZ, SC and WJ. Data collection: SL, AD, JL, LW, LLiu and HH. Data analysis and interpretation: SW, HL. Study guidance: SX, YS, LZL, LL and PC. Manuscript writing and revision: All authors. Final approval of the manuscript: All authors.

## Funding

This study has received funding by the Key-Area Research and Development of Guangdong Province (Grant No. 2020B1111190001), Science and Technology Planning Project of Guangzhou City, China (Grant No. 201907010043) and the National Natural Science Foundation of China (Grant No. 61975244, 82171906, and 81902638).

## Acknowledgments

We also acknowledge the help of English language editing from Editage (www.editage.com).

## Conflict of interest

The authors declare that the research was conducted in the absence of any commercial or financial relationships that could be construed as a potential conflict of interest.

## Publisher’s note

All claims expressed in this article are solely those of the authors and do not necessarily represent those of their affiliated organizations, or those of the publisher, the editors and the reviewers. Any product that may be evaluated in this article, or claim that may be made by its manufacturer, is not guaranteed or endorsed by the publisher.

## Footnotes

1
https://analyzedirect.com/analyzepro/

2
https://github.com/Radiomics/pyradiomics

3
http://www.r-project.org/
