# Supplementary material for: Establishment and validation of novel MRI radiomic feature-based prognostic models to predict progression-free survival in locally advanced rectal cancer
Source: Front Oncol. 2022 Nov 3;12:901287. doi: 10.3389/fonc.2022.901287 (PMC9669703; doi:10.3389/fonc.2022.901287)
Supplement: Supplementary file 1 [file DataSheet_1.docx]

**Supplementary Information**

1. **Description of the LASSO method**

The least absolute shrinkage and selection operator (LASSO) algorithm is a regression analysis method that performs both variable selection and regularization in order to enhance the prediction accuracy and interpretability of the statistical model it produces. The LASSO is a data analysis method that may be applied for biomarker selection in high dimensional data. It was originally proposed for linear regression models to minimize the residual sum of squares, subject to the sum of the absolute value of the coefficients being less than a tuning parameter (λ). The LASSO method was cross-validated to determine an optimal tuning parameter lambda. All features with non-zero coefficients (at the optimal lambda) were selected.

The LASSO method was combined with Cox proportional hazard regression model for survival analysis in this study. We established model with the *cv.glmnet* function in the glmnet package, and the *cv-glmnet* function makes use of cross test to observe model error with different lambda values. We chose the tuning parameters which minimize the model error as the optimal tuning parameter. All features with non-zero coefficients (at the optimal lambda) were selected.

1. **Radiomics score Rad score calculation formula**

**Radscore** = 9.702 * T1C-w_gradient_glszm_SmallAreaEmphasis

+32.55 * T1C-w_log-sigma-3-0-mm-3D_glcm_Imc1

–0.4677 * T1C-w_wavelet-LHH_glszm_SmallAreaHighGrayLevelEmphasis

–0.9219 * T1C-w_wavelet-HHH_glszm_HighGrayLevelZoneEmphasis

–16.89 * T1-w_wavelet-HLH_glszm_SizeZoneNonUniformityNormalized

+4.035 * T1-w_wavelet-HHL_glszm_ZoneEntrop

–81.06 * T2-w_wavelet-HHL_glszm_SizeZoneNonUniformityNormalized

–25.78 * T2-w_wavelet-HHL_glszm_GrayLevelNonUniformityNormalized

+2.788 * T2-w_wavelet-HHL_glszm_ZoneEntropy

+1319 * T2-w_wavelet-HHH_glcm_InverseVariance

+54.53 * short-axis-T2-w_original_glszm_SmallAreaEmphasis

1. **Supplementary Tables**

| **Supplementary table 1** Univariate analysis of clinical predictors for 3-year PFS in the training cohort | | |
| --- | --- | --- |
| Variables | Univariate Cox regression | |
|  | HR(95% CI) | *P* values |
| Clinical T stage |  | 0.393 |
| T2 | 1 (reference) |  |
| T3 | 1.181 (0.156-8.940) |  |
| T4 | 1.732 (0.213-14.100) |  |
| Clinical N stage |  | 0.820 |
| N0 | 1 (reference) |  |
| N1 | 0.545 (0.183-1.622) |  |
| N2 | 1.025 (0.396-2.654) |  |
| TRG |  | 0.087* |
| 1 | 1 (reference) |  |
| 2 | 1.055(0.305-3.650) |  |
| 3 | 1.743(0.573-5.300) |  |
| 4 | 2.824(0.799-9.989) |  |
| CEA |  | 0.074* |
| ≤ 5ng/ml | 1 (reference) |  |
| > 5ng/ml | 2.126 (0.930-4.863) |  |
| CA19-9 |  | 0.062* |
| ≤ 35U/ml | 1 (reference) |  |
| > 35U/ml | 2.438 (0.957-6.211) |  |
| GLO |  | 0.001* |
| 20 to 40g/L | 1 (reference) |  |
| > 40 or < 20g/L | 12.290 (2.777-54.420) |  |
| ypT stage |  | 0.012* |
| T0 | 1 (reference) |  |
| T1 | -0.158(0.088-8.342) |  |
| T2 | 0.530(0.439-6.585) |  |
| T3 | 1.615(1.380-18.308) |  |
| T4 | 0.839(0.235-22.731) |  |
| ypN stage |  | 0.864 |
| N0 | 1 (reference) |  |
| N1 | -0.173(0.250-2.834) |  |
| N2 | 0.617(0.248-13.847) |  |

***Note*:** Hazard ratios estimated by Cox proportional hazards regression. All statistical tests were two-sided. Candidate variables with a *P* value < 0.1 (*) were included in multivariable model analysis.

**Abbreviations**: PFS, progression-free survival; HR, hazard ratio; CI, confidence interval; ypT/N, the pathologic classification after nCRT; TRG, tumor regression grade; CEA, carcinoembryonic antigen; CA19-9, carbohydrate antigen 19-9, GLO, globulin.

| **Supplementary table 2** Multivariate Cox regression results of clinical prognostic models for 3-year PFS in the training cohort | | | | | |
| --- | --- | --- | --- | --- | --- |
| **Models for survival prediction** | | Variables | Coefficient | HR(95% CI) | *P* values* |
| Pre-treatment clinical prognostic models |  | Clinical T stage |  |  |  |
|  | Clinical stage prognostic model | T2 |  |  |  |
|  |  | T3 | 0.048 | 1.050(0.136-8.092) | 0.963 |
|  |  | T4 | 0.458 | 1.580(0.193-12.921) | 0.670 |
|  |  | Clinical N stage |  |  |  |
|  |  | N0 |  |  |  |
|  |  | N1 | -0.656 | 0.519(0.172-1.570) | 0.246 |
|  |  | N2 | -0.046 | 0.955(0.361-2.530) | 0.927 |
|  | PreM | CEA |  |  |  |
|  |  | ≤ 5ng/ml |  |  |  |
|  |  | > 5ng/ml | 0.886 | 2.425(1.034-5.689) | 0.042 |
|  |  | GLO |  |  |  |
|  |  | 20 to 40g/L |  |  |  |
|  |  | > 40 or < 20g/L | 2.832 | 16.987(3.612-79.892) | <0.001 |
| Post-treatment clinical prognostic models | Pathologic stage prognostic model | ypT stage |  |  |  |
|  |  | T0 |  |  |  |
|  |  | T1 | -0.096 | 0.909(0.092-8.939) | 0.935 |
|  |  | T2 | 0.520 | 1.682(0.422-6.706) | 0.461 |
|  |  | T3 | 1.665 | 5.285(1.431-19.514) | 0.013 |
|  |  | T4 | 0.831 | 2.296(0.233-22.618) | 0.476 |
|  |  | ypN stage |  |  |  |
|  |  | N0 |  |  |  |
|  |  | N1 | -0.359 | 0.698(0.204-2.393) | 0.568 |
|  |  | N2 | 0.734 | 2.083(0.261-16.632) | 0.489 |
|  | PostM1 | CEA |  |  |  |
|  |  | ≤ 5ng/ml |  |  |  |
|  |  | > 5ng/ml | 0.693 | 2.000(0.844-4.740) | 0.115 |
|  |  | GLO |  |  |  |
|  |  | 20 to 40g/L |  |  |  |
|  |  | > 40 or < 20g/L | 3.776 | 43.661(7.249-262.980) | <0.001 |
|  |  | ypT stage |  |  |  |
|  |  | T0 |  |  |  |
|  |  | T1 | 0.508 | 1.662(0.153-18.060) | 0.676 |
|  |  | T2 | 0.924 | 2.520(0.576-11.020) | 0.220 |
|  |  | T3 | 2.094 | 8.120(1.824-36.150) | 0.006 |
|  |  | T4 | 1.138 | 3.119(0.275-35.440) | 0.359 |
|  | PostM2 | TRG |  |  |  |
|  |  | 1 |  |  |  |
|  |  | 2 | 0.254 | 1.289(0.353-4.712) | 0.701 |
|  |  | 3 | 0.669 | 1.953(0.599-6.365) | 0.267 |
|  |  | 4 | 1.118 | 3.058(0.843-11.101) | 0.089 |
|  |  | CEA |  |  |  |
|  |  | ≤ 5ng/ml |  |  |  |
|  |  | > 5ng/ml | 0.784 | 2.191(0.934-5.135) | 0.071 |
|  |  | GLO |  |  |  |
|  |  | 20 to 40g/L |  |  |  |
|  |  | > 40 or < 20g/L | 2.963 | 19.360(3.626-103.370) | 0.001 |

***Note*:** Hazard ratios estimated by Cox proportional hazards regression. All statistical tests were two-sided.

**Abbreviations**: PFS: progression-free survival; cTN, the clinical stage prognostic model; PreM, the pre-treatment clinical prognostic model; ypTN, the pathologic stage prognostic model; PostM1, the post-treatment clinical prognostic model; PostM2, the post-treatment clinical prognostic model without pathologic stage; CI, confidence interval; GLO, globulin; TRG, tumor regression grade; CEA, carcinoembryonic antigen; CA19-9, carbohydrate antigen 19-9; HR, Hazard ratios.

| Supplementary table 3 Multivariate cox regression results and description of the radiomics features selected | | | | | |
| --- | --- | --- | --- | --- | --- |
| Image | Filter | Feature Class | Feature | Coefficient | *P* values |
| T1C-w | Gradient | GLSZM‡ | SmallAreaEmphasis | 9.702 | 0.002172 |
| T1C-w | Log | GLCM† | Imc1 | 32.55 | 0.012744 |
| T1C-w | Wavelet(LHH) | GLSZM | SmallAreaHighGrayLevelEmphasis | -0.47 | 0.019692 |
| T1C-w | Wavelet(HHH) | GLSZM | HighGrayLevelZoneEmphasis | -0.92 | 0.002175 |
| T1-w | Wavelet(HLH) | GLSZM | SizeZoneNonUniformityNormalized | -16.9 | 0.045393 |
| T1-w | Wavelet(HHL) | GLSZM | ZoneEntropy | 4.035 | 0.000438 |
| T2-w | Wavelet(HHL) | GLSZM | SizeZoneNonUniformityNormalized | -81.1 | 0.00000341 |
| T2-w | Wavelet(HHL) | GLSZM | GrayLevelNonUniformityNormalized | -25.8 | 0.000381 |
| T2-w | Wavelet(HHL) | GLSZM | ZoneEntropy | 2.788 | 0.005321 |
| T2-w | Wavelet(HHH) | GLCM | InverseVariance | 1319 | 0.0000117 |
| short-axis T2-w | Original | GLSZM | SmallAreaEmphasis | 54.53 | 0.00000347 |
| ‡ Gray Level Size Zone Matrix (GLSZM): A Gray Level Size Zone quantifies gray level zones in an image. A gray level zone is defined as a number of connected voxels that share the same gray level intensity. | | | | | |
| † Gray Level Co-occurrence Matrix (GLCM): A Gray Level Co-occurrence Matrix of size Ng×Ng describes the second-order joint probability function of an image region constrained by the mask and is defined as P(i,j\|δ,θ). | | | | | |
| * The descriptions and feature explanation were downloaded from the official website of "pyradiomics" (https://pyradiomics.readthedocs.io). | | | | | |

| **Supplementary table 4** Corresponding HR and *P* values of clinical predictors when performing multivariable Cox regression using radscore and each of the clinical factors below | | | | | | | | | | |
| --- | --- | --- | --- | --- | --- | --- | --- | --- | --- | --- |
| Clinical factors | HR | *P* values |  | Clinical factors | HR | *P* values |  | Clinical factors | HR | *P* values |
| TRG | 1.480 | 0.082 |  | Chemotherapy | 0.684 | 0.413 |  | Diabetes | 0.855 | 0.804 |
| LDL-C | 0.473 | 0.104 |  | XELOX | 0.916 | 0.455 |  | LDH | 0.772 | 0.805 |
| TC | 3.832 | 0.123 |  | CRE | 1.748 | 0.498 |  | ALB | 0.901 | 0.830 |
| CA19-9 | 2.253 | 0.128 |  | apoB | 0.723 | 0.529 |  | Smoking or not | 0.918 | 0.840 |
| AST | 0.475 | 0.140 |  | GLO | 1.614 | 0.534 |  | apoA | 1.092 | 0.842 |
| ypT | 1.322 | 0.149 |  | BMI | 1.190 | 0.550 |  | IBIL | 1.240 | 0.850 |
| CRP | 1.809 | 0.185 |  | Age | 0.987 | 0.575 |  | MONO | 1.114 | 0.864 |
| CEA | 1.703 | 0.215 |  | WBC | 1.805 | 0.580 |  | GLU | 1.067 | 0.895 |
| ALT | 0.438 | 0.276 |  | PLT | 0.704 | 0.582 |  | TG | 0.946 | 0.908 |
| Clinical N stage | 1.355 | 0.281 |  | Drink or not | 0.773 | 0.646 |  | GGT | 0.949 | 0.935 |
| ALP | 0.326 | 0.288 |  | Clinical T stage | 1.138 | 0.689 |  | LY | 0.960 | 0.957 |
| Gender | 0.595 | 0.328 |  | Hypertension or not | 0.798 | 0.712 |  | BUN | 0.000 | 0.998 |
| NE | 0.511 | 0.380 |  | HGB | 1.167 | 0.727 |  | DBIL | 0.138 | 1.000 |
| RBC | 0.693 | 0.408 |  | ypN | 1.164 | 0.767 |  |  |  |  |

***Note***: Hazard ratios estimated by Cox proportional hazards regression. All statistical tests were two-sided. *P* values for clinical factors except TRG are greater than 0.1. Radiomics signature weaken the discriminatory power of the clinical prognostic indicator including blood test, age, gender, staging, etc.

***Abbreviations:*** TRG, tumor regression grade; LDL-C, low density lipoprotein cholesterol; TC, serum total cholesterol; CA19-9, carbohydrate antigen 19-9; AST, aspartate transaminase; CRP, C-Reactive Protein; CEA, carcinoembryonic antigen; ALT, alanine transaminase; ALP, alkaline phosphatase; NE, neutrocyte count; RBC, red blood cell count; CRE, creatinine; apoB, apolipoprotein B; GLO, globulin; BMI, body mass index; WBC, white blood cell count; PLT, platelet count; HGB, hemoglobin; LDH, lactic dehydrogenase; ALB, albumin; apoA, apolipoprotein A; IBIL, indirect bilirubin; MONO, monocytes count; GLU, blood sugar; TG, triglyeride; GGT, gamma-glutamyl transpeptidase; LY, lymphocyte count; BUN, blood Urea Nitrogen; DBIL, serum direct bilirubin.

1. **Supplementary Figures**


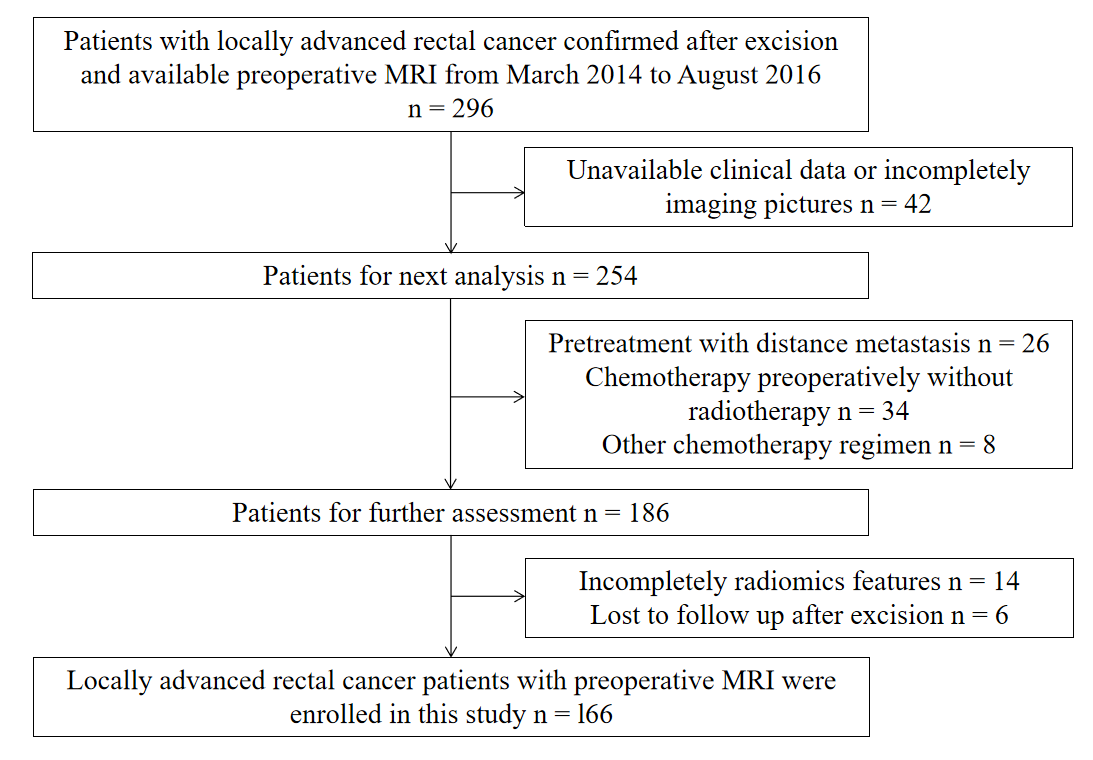


**Supplementary Figure 1** Flowchart summarizes patient enrollment and outcomes.

**
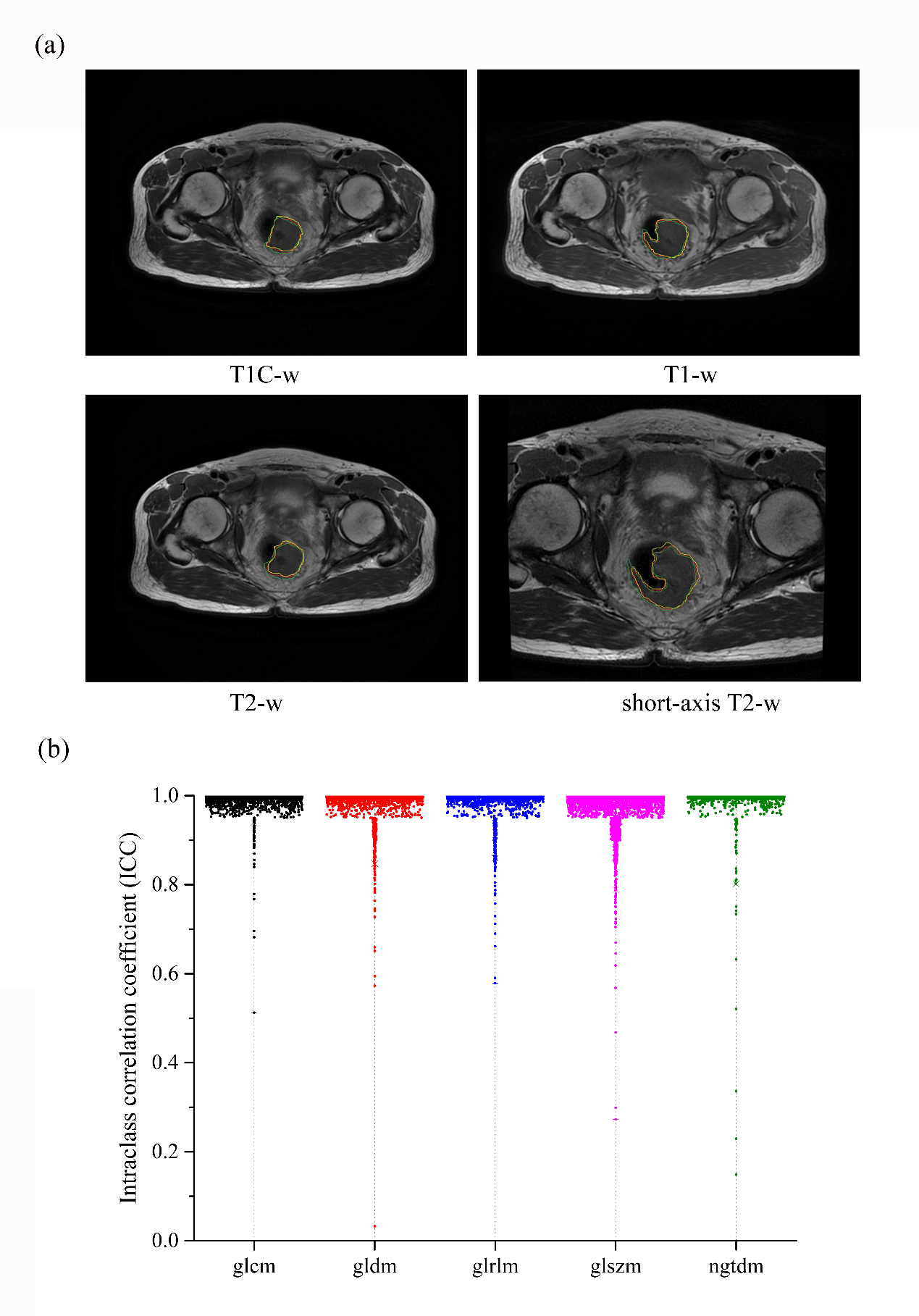
**

**Supplementary Figure2 (a)** An example shows the segmentation results of the same colorectal cancer by different expert radiologists. **(b)** Boxplot of intra-class correlation coefficient of radiomics features for variation in manual segmentations by expert radiologists.

**
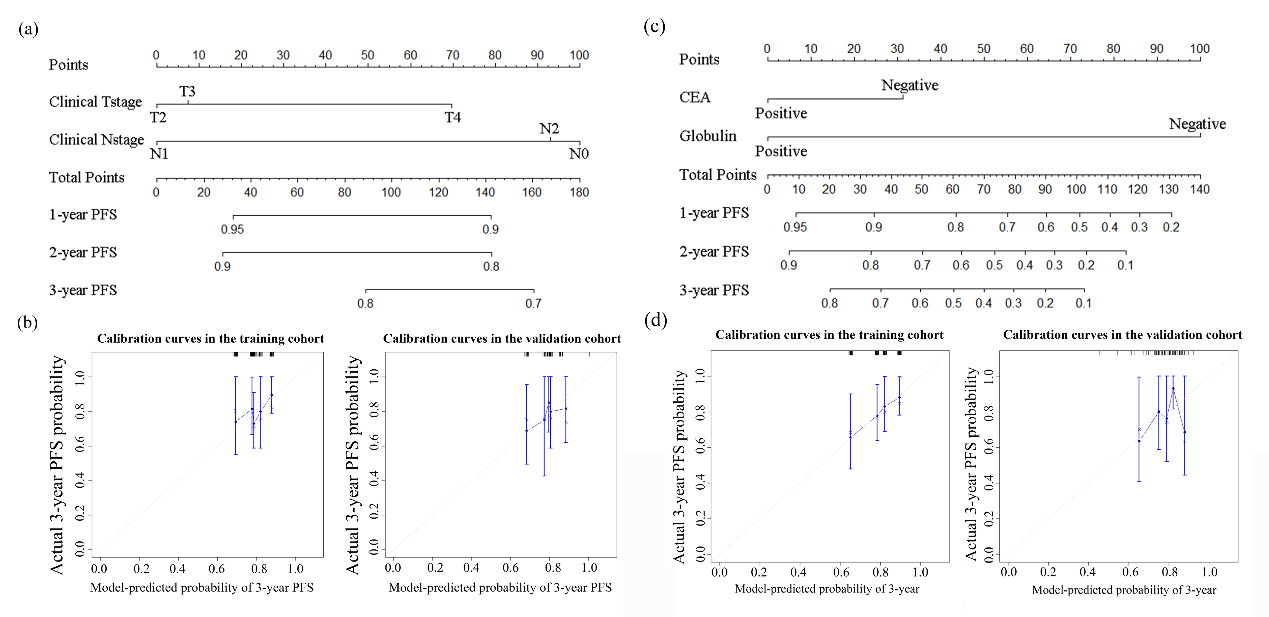
**

**Supplementary Figure 3** Nomogram for 3-year PFS: for **a)** the clinical stage prognostic model and **c)** PreM. Calibration curves for predicting 3-year PFS: for **b)** the clinical stage prognostic model and **d)** PreM in the training cohort and validation cohort.

**Abbreviations**: PFS: progression-free survival; PreM: the pre-treatment clinical prognostic model.


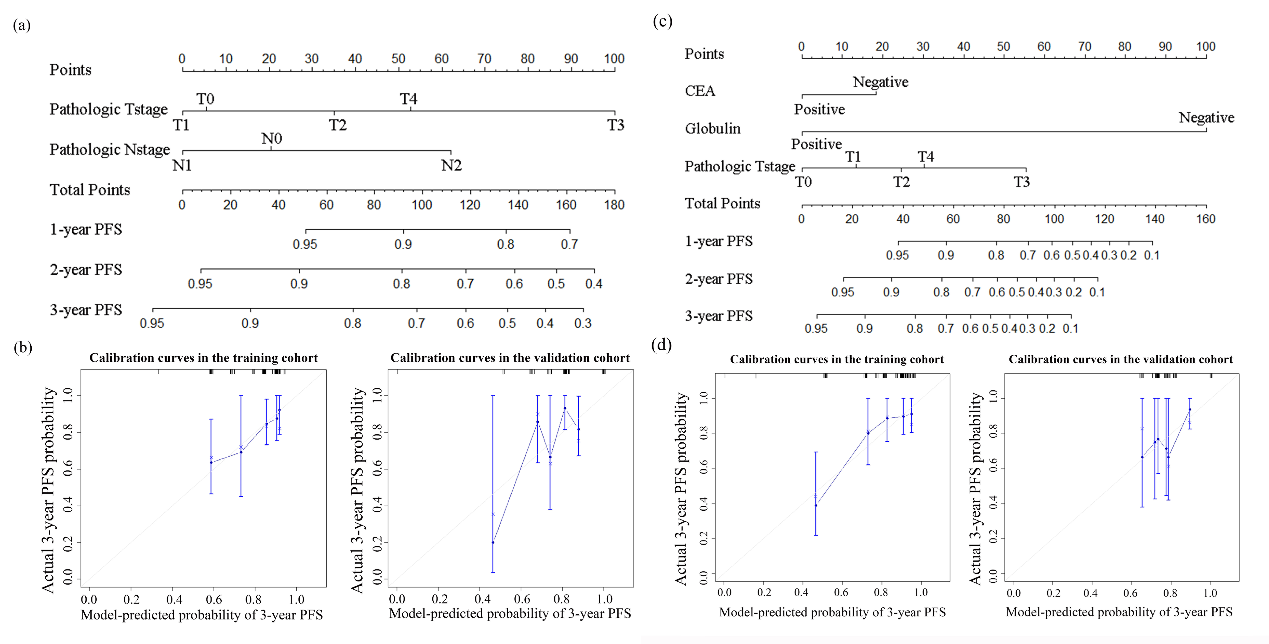


**Supplementary Figure 4** Nomogram for 3-year PFS: for **a)** the pathologic stage prognostic model and **c)** PostM1. Calibration curves for predicting 3-year PFS: for **b)** the pathologic stage prognostic model and **d)** PostM1 in the training cohort and validation cohort. The closer fit to the diagonal dotted line indicates a better assessment.

**Abbreviations**: PFS: progression-free survival; PostM1: the post-treatment clinical prognostic model.


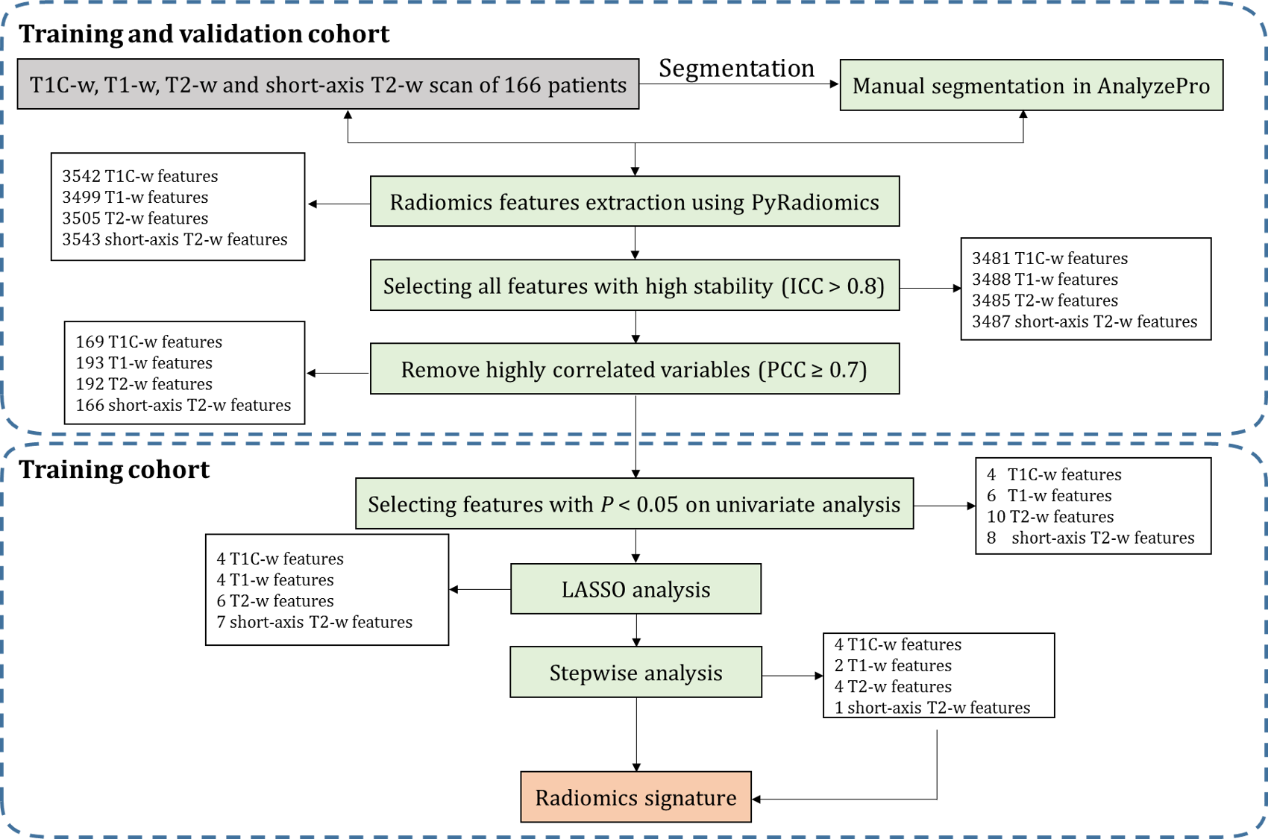


**Supplementary Figure 5** Flowchart implementing the process of radiomics features selecting.

**Abbreviations**: T1C-w, Contrast-enhanced T1-weighted; T1-w, T1-weighted; T2-w, T2-weighted; short-axis T2-w, T2-weighted Field of View; ICC, Inter-class correlation coefficient; PCC, Pearson correlation coefficient; LASSO, Least absolute shrinkage and selection operator.


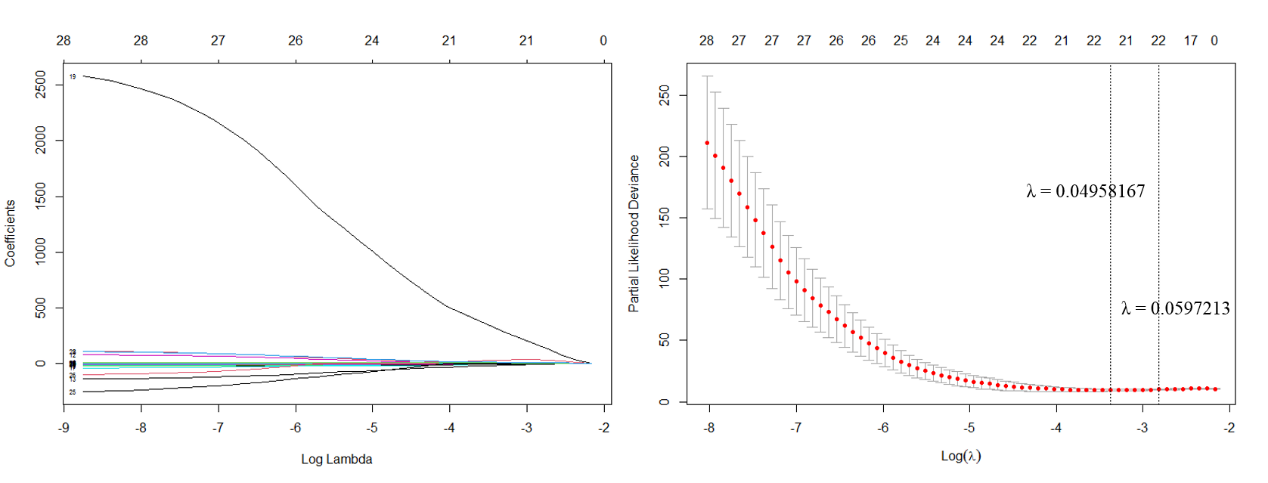


**Supplementary Figure 6** Radiomics feature selection using the least absolute shrinkage and selection operator (LASSO) Cox regression method. Identification of the optimal penalization coefficient lambda (λ) in the LASSO model used 10-fold cross-validation and the minimum criterion. The dotted vertical line was plotted at the value selected using 10-fold cross-validation. The residual sum of squares was minimum when lambda equaled 0.04958167, yielding 11 independent and highly significant features.


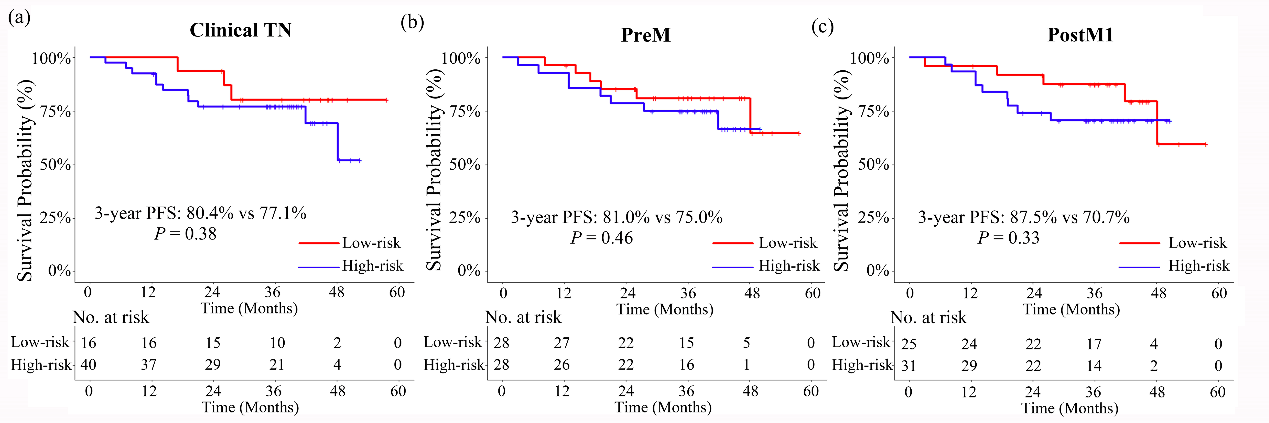


**Supplementary Figure7** Stratified Kaplan-Meier analyses of the prognostic models to estimate PFS in various risk stratification subgroups in the validation cohort. The log-rank test was used to calculate *P* values.

**Abbreviations**: Clinical TN, clinical stage prognostic model; PreM, the pre-treatment clinical prognostic model; PostM1, the post-treatment clinical prognostic model.


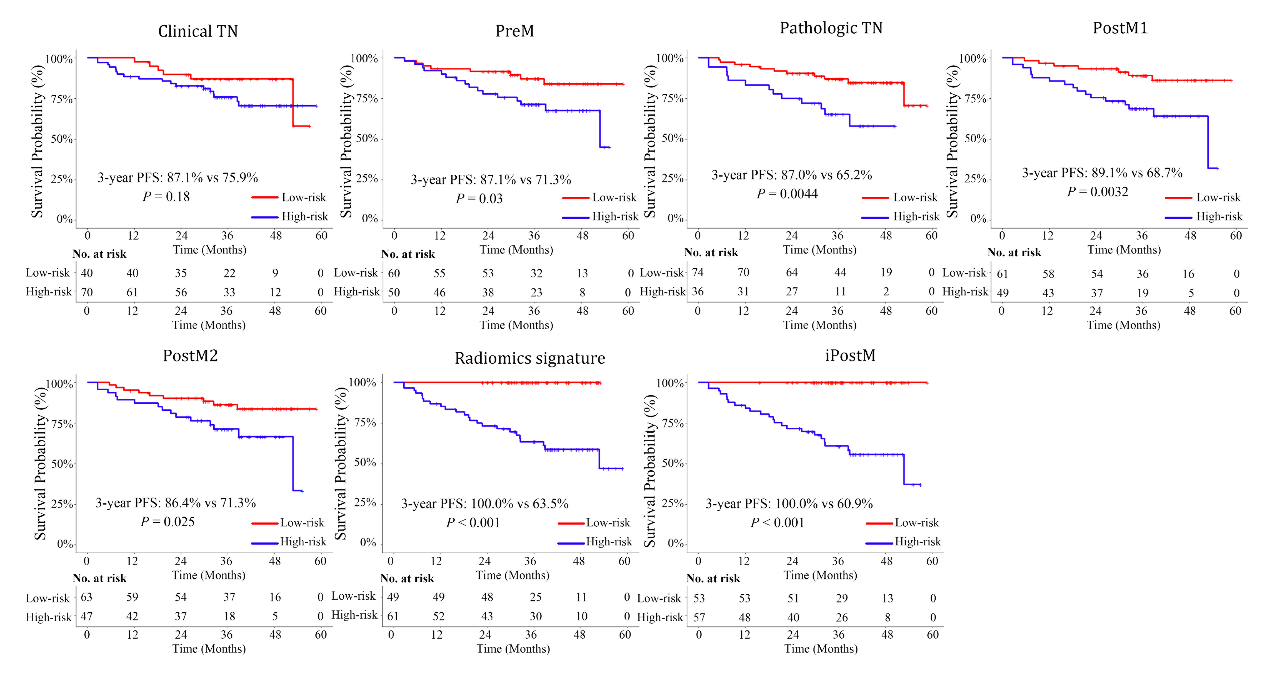


**Supplementary Figure 8** Stratified Kaplan-Meier analyses of the prognostic models to estimate PFS in various risk stratification subgroups in the training cohort. The log-rank test was used to calculate *P* values.

**Abbreviations**: Clinical TN, clinical stage prognostic model; PreM, the pre-treatment clinical prognostic model; Pathologic TN, pathologic stage prognostic model; PostM1, the post-treatment clinical prognostic model; PostM2, the post-treatment clinical prognostic model without pathologic stage; iPostM, the integrated prognostic model combining TRG and radiomics signature.

**
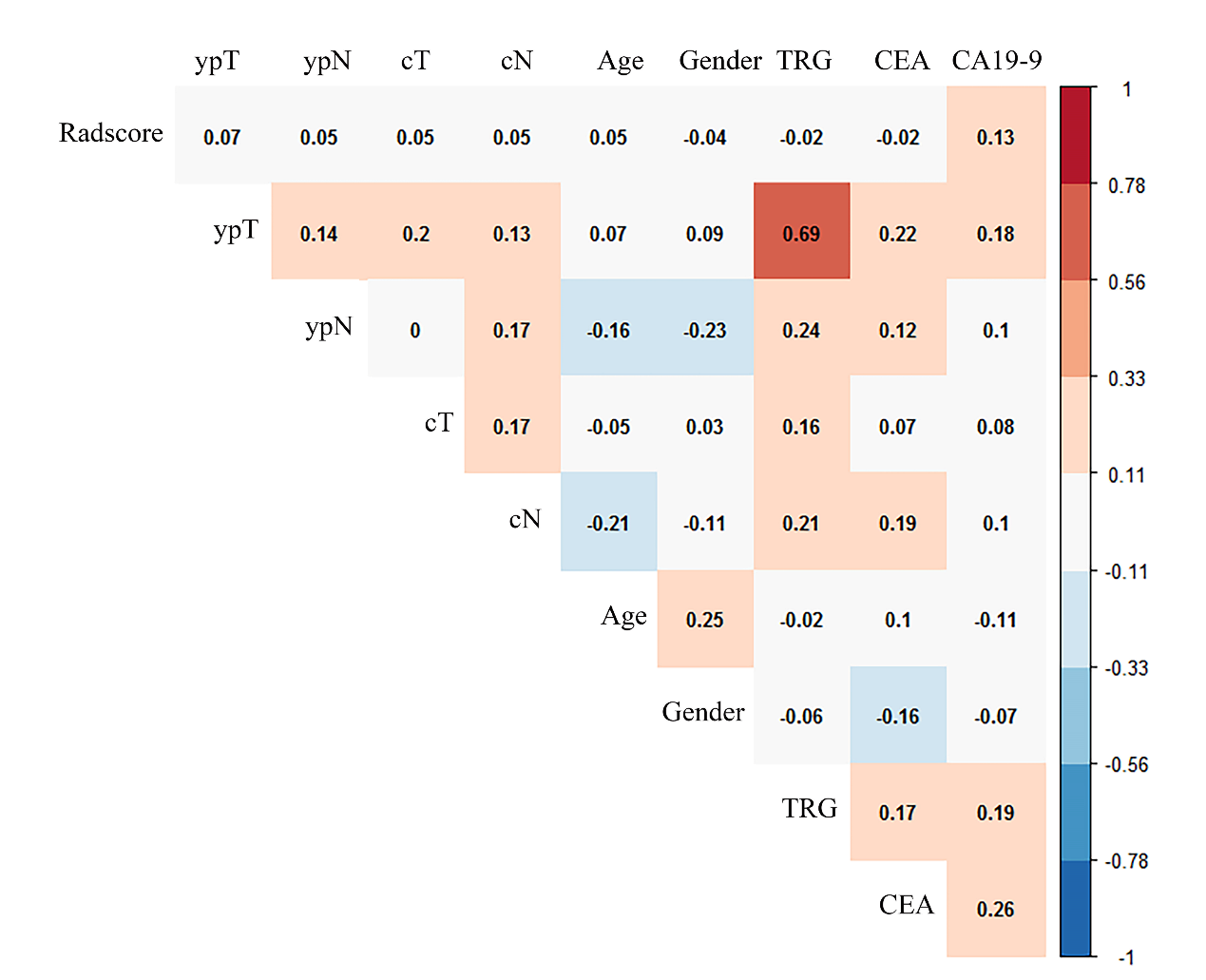
**

**Supplementary Figure9** Heatmap depicting the associations between the radiomics signature and selected clinical factors. Data in the rectangle indicate the Spearman correlation coefficient values.

1. **The parameter settings for image preprocessing and radiomics feature extraction**

***Note:***

- This is our settings used for image processing and feature extraction in this research. Some points in determining better values are added as comments where appropriate.
- For each ROI, seven built-in filters (wavelet, Laplacian of Gaussian, original, square, square root, logarithm, and exponential) were applied and seven feature classes (first order statistics, shape descriptors, and texture classes (including gray level cooccurrence matrix, gray level run length matrix, gray level size zone matrix , gray level dependence matrix, and neighboring gray tone difference matrix) ) were calculated.
- All MRI data were subjected to imaging normalization (the intensity of the image was scaled to 0–100) before feature extraction.
- All MRI data were resampled to the same resolution (3 mm×3mm×3 mm) before feature extraction.
- Extracted using PyRadiomics version: <2.2.0>

***Parameter settings:***

imageType:

Original: {}

LoG:

# Because of resampling to (3, 3, 3), the use of sigmas < 3 mm is not recommended.

sigma: [3.0, 5.0]

Wavelet: {}

Square: {}

SquareRoot: {}

Logarithm: {}

Exponential: {}

Gradient: {}

featureClass:

# redundant Compactness 1, Compactness 2 an Spherical Disproportion features are disabled by default, they can be

# enabled by specifying individual feature names (as is done for glcm) and including them in the list.

shape:

firstorder:

glcm: # Disable SumAverage by specifying all other GLCM features available

- 'Autocorrelation'

- 'JointAverage'

- 'ClusterProminence'

- 'ClusterShade'

- 'ClusterTendency'

- 'Contrast'

- 'Correlation'

- 'DifferenceAverage'

- 'DifferenceEntropy'

- 'DifferenceVariance'

- 'JointEnergy'

- 'JointEntropy'

- 'Imc1'

- 'Imc2'

- 'Idm'

- 'Idmn'

- 'Id'

- 'Idn'

- 'InverseVariance'

- 'MaximumProbability'

- 'SumEntropy'

- 'SumSquares'

glrlm:

glszm:

gldm:

ngtdm:

setting:

normalize: true #set to True to enable normalizing of the image before any resampling

normalizeScale: 100 # This allows you to use more or less the same bin width.

# Resampling:

# Increasing the resampled spacing forces PyRadiomics to look at more coarse textures, which may or

# may not increase accuracy and stability of your extracted features. Using a small spacing in large slices generates

# many interpolated voxels, potentially 'masking' the signal contained in the image.

interpolator: 'sitkBSpline'

resampledPixelSpacing: [3, 3, 3]

# Mask validation:

# correctMask and geometryTolerance are not needed, as both image and mask are resampled, if you expect very small

# masks, consider to enable a size constraint by uncommenting settings below:

#minimumROIDimensions: 2

#minimumROISize: 50

# Image discretization:

# The ideal number of bins is somewhere in the order of 16-128 bins. A possible way to define a good binwidt is to

# extract firstorder:Range from the dataset to analyze, and choose a binwidth so, that range/binwidth remains approximately

# in this range of bins.

binWidth: 25

# first order specific settings:

# When normalizing, gray values below the mean will be negative. Shifting by 300 (3 StdDevs * 100) ensures that the

# majority of voxels is positive (only outliers >3 SD lower than the mean will be negative).

voxelArrayShift: 300

# Misc:

# default label value. Labels can also be defined in the call to featureextractor.execute, as a commandline argument,

# or in a column "Label" in the input csv (batchprocessing)

label: 1
